# 布鲁顿酪氨酸激酶抑制剂治疗B细胞淋巴瘤相关心血管疾病合并症管理专家共识（2023年版）

**DOI:** 10.3760/cma.j.issn.0253-2727.2023.05.002

**Published:** 2023-05

**Authors:** 中国抗癌协会血液肿瘤专业委员会 中华医学会血液学分会 中国慢性淋巴细胞白血病工作组

布鲁顿酪氨酸激酶抑制剂（Bruton tyrosine kinase inhibitor，BTKi）作为治疗B细胞淋巴瘤（B cell lymphoma，BCL）的小分子靶向药物，在多种亚型中被国内外指南推荐。BTKi治疗过程中可能发生心房颤动（atrial fibrillation，AF）、高血压等不良事件，同时心血管疾病（cardiovascular diseases，CVDs）是淋巴瘤患者常见合并症和死亡原因之一。为加强BTKi治疗BCL过程中CVDs管理，减少相关并发症，确保患者临床获益，中国抗癌协会血液肿瘤专业委员会、中华医学会血液学分会及中国慢性淋巴细胞白血病工作组组织血液病学、心脏病学专家，结合国内外最新的权威指南及循证医学证据共同制定了本共识。

一、共识制定方法

1. 共识形成过程：该共识是由中国抗癌协会血液肿瘤专业委员会、中华医学会血液学分会及中国慢性淋巴细胞白血病工作组组成联合工作组制定的，工作组由国内血液病学和心脏病学专家组成。2022年10月线上召开了第一次共识会议，2023年2月在山东济南召开了第二次共识会议。工作组审查并讨论了BTKi治疗BCL相关CVDs合并症治疗的现有证据，并根据现有的临床证据和工作组成员的集体经验形成了共识建议初稿。根据工作组成员充分协商达成的一致意见修订了相关建议，最后形成共识终稿。

2. 文献检索：于2022年3月使用PubMed进行文献检索，检索表达式：（“Bruton's tyrosine kinase inhibitor” OR “BTK inhibitor” OR “BTKi” OR “ibrutinib” OR “acalabrutinib” OR “zanubrutinib” OR “orelabrutinib” OR “pirtobrutinib” OR “Tirabrutinib”）AND（“hypertension” OR“atrial fibrillation” OR “AF” OR “sudden cardiac death” OR “SCD” OR “ventricular arrhythmia” OR “VA” OR “cardiovascular diseases” OR “CVDs” OR “heart failure” OR “HF” OR “coronary atherosclerotic cardiopathy”），限定为英文出版物。

二、启动BTKi前的评估

开始BTKi治疗前，建议对心血管系统进行全面评估及检查：①询问病史及合并用药，包括心脏瓣膜病、室性心律失常（ventricular arrhythmia，VA）、缺血性心脏病史、心力衰竭（heart failure，HF）或左心室功能障碍/射血分数降低病史；②询问是否存在CVDs风险因素，包括糖尿病、肥胖、高血压、血脂异常、慢性肾病；③测量血压及检查心电图（electrocardiograph，ECG），优选24 h动态心电图；④对于存在CVDs高风险或已有AF、HF、糖尿病、冠状动脉疾病和高血压的患者[1]，推荐超声心动图及基线心脏生物标志物［利钠钛（BNP或NT-proBNP）、肌钙蛋白[2]］检查；⑤对于有AF病史的患者，建议转诊至心内科进行心血管的可逆危险因素管理优化，讨论适合的抗凝治疗来预防卒中和控制症状。心血管病高危风险并不是BTKi治疗的禁忌，但存在下列CVDs的患者不宜选择BTKi治疗：有VA病史，重度未控制的高血压［收缩压≥180 mm Hg和（或）舒张压≥120 mm Hg］，重度或未受控制的心力衰竭［左室射血分数（LVEF）<40％］。

三、BTKi治疗BCL相关CVDs合并症管理

1. 高血压：BTKi治疗中可新出现高血压或原有高血压恶化，既往伊布替尼的临床研究中高血压发生率为7％～30％，真实世界中发生率可达35％～78％，半数患者需药物治疗[3]–[5]。

有高血压病史的患者，建议在启动BTKi治疗前完善基线评估及检查，根据心血管病风险分层优化血压管理[6]，BTKi治疗可与降压治疗同时进行，降压目标、抗高血压和抗肿瘤治疗方案应综合多学科团队（MDT）意见。

建议对所有启动BTKi治疗的患者进行高血压相关教育并在治疗过程中持续监测血压［推荐家庭血压监测（HBPM）或动态血压监测（ABPM）］，监测频率为前6个月内至少每周一次，之后每月一次。出现高血压后启动降压治疗可降低主要心血管不良事件（MACEs）风险[3]，主要的血压管理建议包括：①每次血液科就诊时测量血压。收缩压>140 mm Hg和（或）舒张压>90 mm Hg的患者应进行ABPM/HBPM[1]。②一线治疗建议使用血管紧张素受体阻断剂（ARB）、钙离子拮抗剂、利尿剂、β受体阻滞剂。血管紧张素转换酶抑制剂（ACEi）因与伊布替尼同时使用可能增加室性心动过速和心源性猝死（SCD）的风险，应谨慎使用，治疗前已使用ACEi者在启动BTKi时需考虑更换其他药物[7]。③二线降压策略包括卡维地洛、螺内酯、肼屈嗪和口服硝酸酯类药物。对于不能耐受β受体阻滞剂的高血压合并AF患者，如果LVEF超过50％，可考虑二氢吡啶类钙通道拮抗剂；如果LVEF<50％，可以使用氨氯地平，但不推荐硝苯地平。存在液体潴留的患者可使用利尿剂[8]。④通过积极的血压管理可避免BTKi剂量调整；如发生3级或4级（CTCAE分级）高血压，则需中断BTKi治疗，并在不良反应消失后进行剂量调整（表1、2）。高血压药物与BTKi的相互作用见表3。

**表1:** 发生3级及以上高血压或心脏疾病时新一代布鲁顿酪氨酸激酶抑制剂药物剂量调整

不良反应发生次数	泽布替尼不良反应消失后的剂量调整	奥布替尼不良反应消失后的剂量调整
第1次	重新用药，160 mg，每日2次	重新用药，150 mg，每日1次（14 d内恢复）；重新用药，150 mg或100 mg，每日1次（14 d后恢复）
第2次	重新用药，80 mg，每日2次	重新用药，100 mg，每日1次（14 d内恢复）；重新用药，100 mg或50 mg，每日1次（14 d后恢复）
第3次	重新用药，80 mg，每日1次	重新用药，50 mg，每日1次（14 d内恢复）；重新用药，50 mg，每日1次，或停用本品（14 d后恢复）
第4次	停用本品	停用本品

**表2:** 发生不良反应后第一代布鲁顿酪氨酸激酶抑制剂伊布替尼的剂量调整

不良反应	不良反应发生次数	MCL不良反应消失后的剂量调整	CLL/SLL/WM不良反应消失后的剂量调整
2级心力衰竭	第1次	重新用药，420 mg，每日1次	重新用药，280 mg，每日1次
	第2次	重新用药，280 mg，每日1次	重新用药，140 mg，每日1次
	第3次	停用本品	停用本品
3级心律失常	第1次	重新用药，420 mg，每日1次	重新用药，280 mg，每日1次
	第2次	停用本品	停用本品
3级/4级心力衰竭或4级心律失常	第1次	停用本品	停用本品
3级及以上高血压或其他心脏疾病	第1次	重新用药，560 mg，每日1次	重新用药，420 mg，每日1次
	第2次	重新用药，420 mg，每日1次	重新用药，280 mg，每日1次
	第3次	重新用药，280 mg，每日1次	重新用药，140 mg，每日1次
	第4次	停用本品	停用本品

**注** MCL：套细胞淋巴瘤；CLL：慢性淋巴细胞白血病；SLL：小淋巴细胞淋巴瘤；WM：华氏巨球蛋白血症

**表3:** 心血管疾病常见用药与BTK抑制剂（BTKi）合并用药时的注意事项

药物类别	药物名称	与BTKi是否有相互作用	是否可以合用	解释
利尿剂	呋塞米、托拉塞米、阿米洛利	否	是	−
ACEi类	卡托普利、依那普利、贝纳普利	否	谨慎	合用可能会增加室性心动过速和心源性猝死的风险
ARB类	缬沙坦、氯沙坦、坎地沙坦	否	是	−
β受体阻滞剂	美托洛尔、比索洛尔、拉贝洛尔	否	是	−
钙通道拮抗剂	氨氯地平等二氢吡啶类	否	是	−
中效强心苷类药物	地高辛	否	谨慎	地高辛为治疗指数狭窄的口服P-糖蛋白底物，当与BTKi联合给药时，其浓度可能会升高，BTKi给药前后至少6 h内不应使用
抗凝药物	华法林等维生素K拮抗剂	否	否	同时给药可能显著增加出血风险
	DOAC（阿哌沙班、利伐沙班、艾多沙班、达比加群酯）	否	是	共同给药时应密切监测出血迹象
抗血小板药物	阿司匹林、氯吡格雷、替格瑞洛	否	是	共同给药时应密切监测出血迹象
硝酸脂类药物	硝酸异山梨酯	否	是	−
他汀类降胆固醇药物	瑞舒伐他汀	是	是	与泽布替尼联合和使用时，未观察到瑞舒伐他汀的药代动力学有显著临床差异
		是	谨慎	瑞舒伐他汀是经BCRP介导的肝脏外排药物，伊布替尼可全身性抑制BCRP，可增加瑞舒伐他汀暴露量
血管紧张素受体-脑啡肽酶抑制剂	沙库巴曲缬沙坦钠	否	是	−
盐皮质激素受体阻断剂	螺内酯、依普利酮	否	是	−
If通道抑制剂	伊伐布雷定	否	是	−
抗心律失常药物	地尔硫卓、维拉帕米	是	合用需调整BTKi剂量	地尔硫卓、维拉帕米是CYP3A4中效抑制剂
	胺碘酮	是	合用需调整BTKi剂量	胺碘酮为CYP3A4中效抑制剂
	决奈达隆	是	合用需调整BTKi剂量	决奈达隆为CYP3A4中效抑制剂
	氟卡尼	否	是	−
	利多卡因	否	是	−
	美西律	否	是	−
	普罗帕酮	否	是	−
	奎尼丁	是	谨慎	奎尼丁为治疗指数狭窄的口服P-糖蛋白底物，当与BTKi联合给药时，其浓度可能会升高
晚钠电流抑制剂	雷诺嗪	是	是	雷诺嗪为CYP3A4弱抑制剂

**注** BTKi与心血管疾病常用药物之间通过CYP3A4和糖蛋白途径发生药物相互作用，应避免与CYP3A4强效/中效诱导剂、强效/中效抑制剂合用（泽布替尼可减低剂量后与中效或强效抑制剂合用）。ACEi：血管紧张素转换酶抑制剂；ARB：血管紧张素受体阻断剂。更详细的信息可查看https://cancer-druginteractions.org/checker##table-view-wrap

2. AF：AF是最常见的心律失常，且发生率随年龄增长而增加[9]。BTKi治疗期间可能新发AF或使原有AF恶化。伊布替尼治疗期间AF的发生率为4％～10％，真实世界因AF导致的伊布替尼停药率为12％～25％[10]。因此，BTKi治疗中AF的管理与患者的临床获益及生活质量密切相关。

既往AF史并非BTKi治疗的禁忌证。有持续性AF病史的患者应在启动治疗前通过MDT讨论BCL与AF的优化治疗方案，包括是否可启动BTKi治疗、启动前是否可尝试复律及调整抗心律失常药物、卒中/出血风险评估及抗血小板、抗凝药物调整，对合并疾病如甲状腺疾病、糖尿病等的危险因素进行管理等[11]。若AF合并反复失代偿性充血性心力衰竭（CHF），近期合并急性冠状动脉综合征或不可控的出血史，不宜选择BTKi而应考虑其他替代治疗方案[1]。

建议对所有启动BTKi治疗的患者进行AF相关风险及心律监测的教育，如出现胸闷、心悸、呼吸困难及晕厥等症状，及时就诊。BTKi治疗的前12个月，建议患者每次就诊时通过ECG或使用可穿戴心电监测设备增加对AF的筛查，之后至少每3至6个月进行ECG。建议参考《2020 ESC心房颤动诊断和管理指南》的ABC路径管理BTKi相关AF[8],[12]，如表4所示。

**表4:** 布鲁顿酪氨酸激酶抑制剂（BTKi）治疗期间心房颤动（AF）管理建议

ABC路径管理	治疗建议
“A”，即预防卒中	①CHA2DS2-VASc评分（表5）0分（男性）或1分（女性）的患者，继续BTKi治疗，无需抗凝治疗[5]，1分（男性）或2分（女性）可以考虑抗凝治疗。
	②CHA2DS2-VASc评分≥2分（男性）或≥3分（女性）患者建议抗凝治疗[13]，不推荐维生素K拮抗剂如华法林，建议选择新型口服抗凝剂（NOAC），如利伐沙班、阿哌沙班、艾多沙班、达比加群酯，并考虑与BTKi的药物相互作用（表3），避免同时服用维生素E、鱼油等非处方补充剂[14]。
	③使用HAS-BLED量表（表6）评估出血风险，评分≥3分出血风险升高，但非抗凝禁忌，可考虑在标准剂量BTKi治疗期间降低NOAC剂量[2]；注意筛查并纠正增加出血的可逆因素如高血压、过度饮酒或同时服用非甾体类抗炎药[2]；每次就诊时动态评估出血风险，对有出血表现者加强监测并纠正可逆因素，发生大出血的患者建议停止抗凝并调整BTKi剂量[15]。
“B”，即症状控制	①血流动力学稳定者，首选β受体阻滞剂控制心率，无需调整BTKi剂量。
	②β受体阻滞剂无法达到最佳控制的快速AF，BTKi减量。
	③如新发的有症状的3级AF或心力衰竭（HF）等血流动力学不稳定，立即停用BTKi直至稳定，多学科团队（MDT）评估是否重启BTKi。
	④长期BTKi治疗下的心律控制，如出现血流动力学不稳定的紧急情况，可采用电复律或药物复律，但需注意常用药物胺碘酮与BTKi的相互作用，减少BTKi剂量[16]；如出现药物无法控制的症状性AF且B细胞淋巴瘤预计生存超过一年，MDT评估是否导管射频消融[8]。
“C”，即心血管危险因素和合并症风险管理	需重视预防和管理HF，并管理其他相关心血管疾病的危险因素和合并症[2]。

**表5:** CHA_2_DS_2_-VASc风险评分

缩略词	风险因素	评分
C	充血性心力衰竭（或左心室功能不全）	1
H	高血压	1
A2	年龄≥75岁	2
D	糖尿病	1
S2	卒中或短暂性脑缺血发作或血栓栓塞	2
V	血管疾病（既往的心肌梗死、外周动脉疾病和主动脉斑块）	1
A	年龄65～74岁	1
Sc	性别（女性）	1
	满分	9

**表6:** HAS-BLED风险评分

缩略词	临床特征	评分
H	高血压^a^	1
A	肾功能或肝功能异常（各1分）^b^	1～2
S	卒中史	1
B	出血^c^	1
L	国际标准化比值易波动^d^	1
E	老年（年龄>65岁）	1
D	药物^e^或嗜酒（各1分）	1～2

**注** ^a^高血压定义为收缩压>160 mm Hg（1 mm Hg＝0.133 kPa）；^b^肾功能异常定义为慢性透析或肾移植或血清肌酐≥200 µmol/L；肝功能异常定义为慢性肝病（肝纤维化）或胆红素>2倍正常上限，丙氨酸氨基转移酶>3倍正常上限；^c^出血指既往出血史和（或）出血倾向；^d^国际标准化比值易波动指该值不稳定，在治疗窗内的时间<60％；^e^药物指合并应用抗血小板药物或非甾体类抗炎药。最高评分：9分；高风险（≥4分）：年出血风险≥8％；中度风险（2～3分）：年出血风险2％～4％；低风险评分为0；1分：年出血风险<2％

3. VA：有VA病史和心脏骤停史的患者应避免使用BTKi尤其是伊布替尼。对于既往有VA、SCD相关的结构性心肌病、遗传性心律失常综合征（如长QT综合征、Brugada综合征）或遗传性心肌病家族史的患者，应对BTKi的治疗获益进行评估，必要时考虑替代性治疗[8]。患者接受BTKi治疗出现VA的管理及SCD的预防建议有：①QTc延长（>480 ms）者应持续动态监测并参考2022年ESC肿瘤心脏病指南进行干预；②出现晕厥、头晕或心悸症状的患者应立即行ECG、超声心动图和心律监测[8]；③发生特发性VA的患者立即停止BTKi，转诊至心内科以排除潜在心脏疾病[8]；可逆性病因导致VA的患者，纠正病因后可继续应用BTKi，但需密切监测；④注意纠正电解质紊乱，尤其是低钾血症[17]–[18]。BTKi和抗心律失常药物之间需考虑药物的相互作用并进行剂量调整（表3）。

4. 冠状动脉粥样硬化性心脏病：稳定性冠状动脉性心脏病不影响启动BTKi，但需注意合并用药。缓解症状以及改善缺血的药物包括硝酸脂类、β受体阻滞剂、钙通道拮抗剂以及其他（如曲美他嗪、尼可地尔和伊伐布雷定）；改善预后的药物包括抗血小板药物、他汀类药物、β受体阻滞剂、ACEi或ARB。ACEi应谨慎使用，具体见表3。对于启动治疗前曾接受经皮冠状动脉介入术（PCI）而进行双联抗血小板治疗（DAPT）的患者如选择BTKi，需经MDT讨论后平衡DAPT持续时间以及BTKi启动时机，短程DAPT应延迟BTKi治疗，当病情需延长DAPT时则应选择其他抗肿瘤药物。

BTKi治疗过程中进行冠状动脉性心脏病的治疗，建议根据2022年ESC肿瘤心脏病指南并经MDT讨论。对于需接受PCI治疗的患者，DAPT的持续时间应尽可能缩短（1～3个月），期间可中断BTKi治疗；对于同时接受抗凝和抗血小板治疗的患者，短期三联抗血栓治疗（最多住院1周并中断BTKi治疗）后，新型口服抗凝剂（NOAC）和单一口服抗血小板药物（优选氯吡格雷）是默认策略；对于仅接受抗凝治疗患者，通过评估获益及风险，可考虑NOAC单药和BTKi同时使用。

5. CHF：CHF的管理可参考2022年AHA/ACC/HFSA指南，指南建议，LVEF≤40％的心衰患者可接受β受体阻滞剂、盐皮质激素受体阻断剂、地高辛、ACEi、ARB及血管紧张素受体-脑啡肽酶抑制剂（ARNi）、钠-葡萄糖协同转运蛋白抑制剂、袢利尿剂等药物治疗，ACEi应谨慎使用。NYHA分级[19]Ⅲ～Ⅳ级CHF患者避免使用BTKi；Ⅰ～Ⅱ级患者经MDT评估是否适合BTKi治疗并优先推荐第二代BTKi。治疗期间建议每日监测体重并控制钠摄入量低于2 g；至少每3个月心脏科随访；每6至12个月评估肾功能及左心室收缩功能[1]。BTKi治疗期间新发CHF应立即停用BTKi，可耐受患者应用ARB或ARNi加β受体阻滞剂。

四、BTKi选择建议

既往研究提示，第一代BTKi伊布替尼相关≥3级AF的发生率随着时间的推移而降低，而高血压累积发生率随时间推移升高[1]。第二代BTKi具有更高的选择性和更少的脱靶效应，ELEVATE RR[20]和ASPEN[21]研究的中位随访时间分别为40.9个月和44个月，与伊布替尼相比，阿可替尼所有级别的AF及高血压发生率均有所降低，泽布替尼所有级别AF、≥3级AF和高血压发生率均有所降低。ALPINE[22]研究中位随访24.2个月时，泽布替尼的AF、心房扑动发生率分别较伊布替尼降低4.6％和12％，高血压发生率与伊布替尼相当，严重心脏不良事件及相关死亡发生率均更低。在阿可替尼、泽布替尼治疗伊布替尼不耐受患者的研究中，应用第二代BTKi后再次发生AF的发生率及级别均降低。此外，研究报道，奥布替尼及非共价Pirtobrutinib的AF及高血压发生率低[23]–[24]，但尚无与其他BTKi头对头的研究。意大利AF评分（AF score）系统是目前少有的预测慢性淋巴细胞白血病AF风险的评估系统，其风险因素包括：年龄≥65岁（1分）、男性（1分）、心脏瓣膜病（2分）、心脏病史（3分）、甲状腺功能低下/甲状腺功能亢进（2分）、慢性肺部疾病（1分）、2型糖尿病（1分）、≥3级感染（1分），其中评分≥5分、3～4分、1～2分、0分患者接受伊布替尼治疗2年的AF风险分别为40％、17％、5％、0。专家组推荐应用该系统识别接受BTKi治疗患者发生AF的风险[25]，高风险者优先考虑第二代BTKi或其他治疗。如果BTKi治疗期间出现CVDs，建议根据CTCAE 5.0版[26]进行严重程度分级，并按照药物说明书进行剂量调整，具体建议参考表1和表2。当伊布替尼治疗期间发生不能耐受的心脏不良事件，可考虑应用第二代BTKi治疗[27]–[28]。

五、总结

随着在BCL中的循证医学证据增加，BTKi临床应用愈加广泛。推荐各中心CVDs专家作为淋巴瘤多学科诊疗团队成员参与患者的长程随访。血液病学、心血管病学、药理学等多学科模式全程参与患者CVDs的规范管理，有利于制定个性化的、风险适应性强的预防与治疗策略，减少相关并发症，避免患者病情恶化或治疗中断，改善患者预后及生存质量。随着预测工具及积分系统的开发、移动或可穿戴设备的推广应用以及MDT规范管理模式的深入，本共识将不断完善、更新。
